# New observation on *Corynetis* from the early Cambrian Guanshan Biota reflect burrowing life

**DOI:** 10.1098/rsos.251357

**Published:** 2025-10-08

**Authors:** Chunxiao Liu, Jianni Liu

**Affiliations:** ^1^ State Laboratory of Continental Evolution and Early Life, Shaanxi Key Laboratory of Early Life and Environments, Xi'an Key Laboratory of Paleo-bioinformatics, Department of Geology, Northwest University, Xi'an 710069, People's Republic of China; ^2^ Interdisciplinary Research Institute of Advanced Intelligent Equipment, Xihua University, Chengdu 610039, People's Republic of China

**Keywords:** Cambrian, Guanshan Biota, spiny ornamentation, ecology of priapulids

## Abstract

Priapulids demonstrated greater disparity among anatomical morphology and played a pivotal role in early Cambrian marine ecosystems. This disparity appeared in ornamented cuticle and showed adaptability to diverse ecological patterns, which lack detailed investigations. Our study focuses on *Corynetis* from the Guanshan Biota, a poorly understood priapulid taxon with spiny ornamentations, including two species: *Corynetis brevis* and *Corynetis fortis*. Significantly, *Corynetis brevis* is documented within the Guanshan Biota for the first time. A new discovery is the identification of circumoral crown, a novel scalids that encircles the mouth and comprises two rows of eight scalids each, suggesting a sensory capability. In addition, comparative analysis of the terminal trunk spines reveals subtle yet important morphological differences between the two species. This distinction likely reflects differing anchoring strategies, terminal trunk specialization in *Corynetis brevis* versus whole trunk utilization in *Corynetis fortis*, which form single-anchor modes and likely facilitate rapid withdrawal in burrow. As well as the specialized coronal spines that grow faster than other structures, its sensory function can enhance predation. Ultimately, palaeoecological reconstruction suggests that *Corynetis* was a solitary organism with carnivorous habits, predominantly dwelling within a burrow.

## 1. Introduction

Priapulida, a phylum belonging to the Ecdysozoa [1–7], is a clade of vermiform animals inhabiting marine ecosystems. In modern oceans, only the order Priapulomorpha persists, represented by a mere 7 genera and 22 species [7–9]. These extant priapulids form a small relict group. In stark contrast, the Cambrian shallow marine ecosystem [10–13], especially in the Cambrian Series 2 and Series 3, hosted a plethora of ancient priapulids exhibiting abundant quantities and disparate morphologies [14–20]. These ancient priapulids left behind diverse palaeoecological patterns, a record marked by endobenthic burrowing and epibenthic peristalsis [21–24]. Research on functional morphology and ecological adaptations of fossil priapulids is of great significance for revealing the ecological landscape of early animal communities and reconstructing the evolution of early marine ecosystems.

The earliest reliable record of priapulids is *Treptichnus pedum* at the Precambrian-Cambrian boundary [25]. By the early Cambrian (Stage 3, Series 2), priapulids had already undergone an initial radiation [18], with corresponding soft-bodied fossils preserved in the renowned Chengjiang Biota [26–31]. As the further evolution, a wealth of exquisitely preserved fossils emerged in other Burgess Shale-type Lagerstätten in the Cambrian, including the Guanshan Biota (Stage 4, Series 2) [10,32–36], the Kaili Biota (Stage 5, Series 3) [18,37,38] and the Burgess Shale Biota (Stage 5, Series 3) [7,39–42]. The Guanshan Biota is stratigraphically positioned between the younger Chengjiang Biota and the older, roughly coeval Kaili Biota and Burgess Shale Biota [13,34,43]. To date, 10 genera and 10 species of priapulids have been reported from there: *Guanduscolex minor* [10,32], *Yunnanoscolex magnus* [10], *Wudingscolex sapushanensis* [10], *Paramaotianshania zijunia* [10], *Palaeoscolex xinglongensis* [34], *Mafangscolex* cf. *yunnanensis* [34], *Corynetis fortis*, *Xiaoheiqingella* sp. [33], *Sicyophorus* sp. [35] and ?*Eximipriapulus* sp. [36]. It offers a crucial window into priapulid morphological evolution.

**Figure 1.**
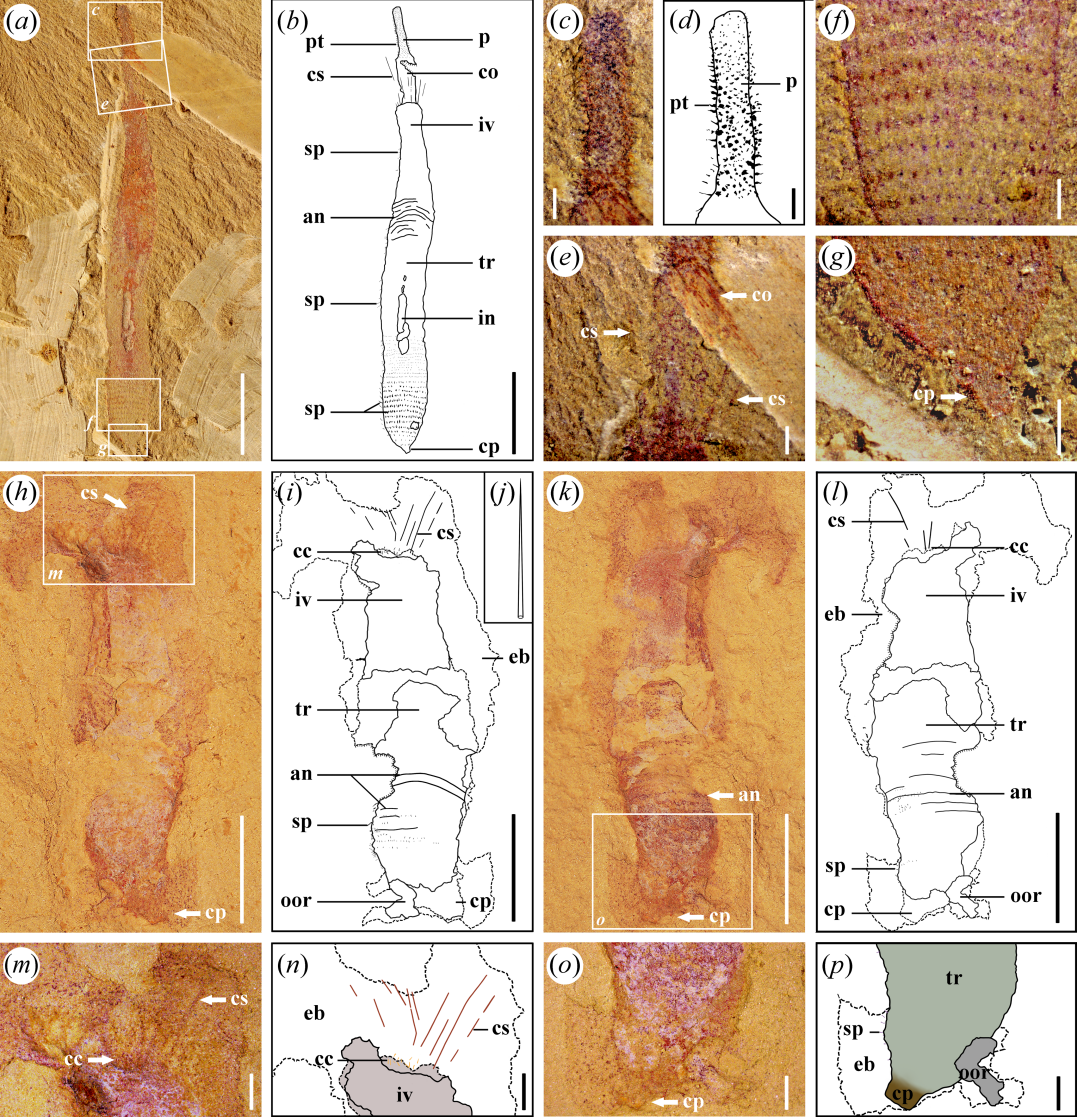
*Corynetis brevis* from the Chengjiang Biota and Guanshan Biota. (*a−g*) JSWB−0068, *Corynetis brevis* of the Chengjiang Biota (provided by Professor Jian Han); (*a*) Part, specimen has typical structures such as pharyngeal teeth (pt), collar (co), coronal spines (cs), spines (sp) and everted caudal projection (cp); (*b*) Illustration of part; (*c*) Enlarged image reflects the pharynx within the boxed area in (*a*), showing pharyngeal teeth (pt) on it; (*d*) Illustration of the pharynx; (*e*) Enlarged image reflects the collar (co) and coronal spines (cs) within the boxed area in (*a*); (*f*) Enlarged image reflects the spines (sp) on the trunk within the boxed area in (*a*); (*g*) Enlarged image reflects the caudal projection (cp) within the boxed area in (*a*); (*h-p*) LBS−541, *Corynetis brevis* of the Guanshan Biota; (*h*) Part, specimen has typical structures such as coronal spines (cs), circumoral crown (cc), spines (sp), and everted caudal projection (cp); (*i*) Illustration of part; (*j*) Illustration of the coronal spine (cs); (*k*) Counterpart; (*l*) Illustration of counterpart; (*m*) Enlarged image reflects the scalids within the boxed area in (*h*), including coronal spines (cs) and circumoral crown (cc); (*n*) Illustration of (*m*); (*o*) Enlarged image of terminal trunk within the boxed area in (*k*), presenting an everted caudal projection (cp); (*p*) Illustration of (*o*). Abbreviations: an, annulations; cc, circumoral crown; co, collar; cp, caudal projection; cs, coronal spines; eb, exceeded body fluid; in, intestine; iv, introvert; oor, other organisms; p, pharynx; pt, pharyngeal teeth; sp, spines; tr, trunk. Scale bars: (*a, b, h, i, k, l*), 5 mm; (*c−g*), 0.5 mm; (*m−p*), 1 mm.

Typically, fossil priapulids preserve disparate cuticular structures [5,18], including pharyngeal teeth, scalids, sclerites [17,27,32,34,43–49], papillaes [14,50], spines [10,28,51–53] and reticulated ornament [5,47]. These tiny cuticular structures are crucial for priapulid taxonomy [5,18]. In this study, we focus on *Corynetis*, a morphologically distinct priapulid genus, characterized by a clubbed shape and spinose body [10,28,51]. The spiny ornamentations of this genus consist of pharyngeal teeth and scalids on the proboscis, as well as spines on the trunk, which are the major factors for the research of *Corynetis*. To date, two species of *Corynetis* have been described: *Corynetis brevis*, first reported from the Chengjiang Biota (figure 1*a*–*g*) in 1999 [28,51], and *Corynetis fortis*, later identified in the Guanshan Biota in 2012 [10]. In the analysis of anatomical and functional morphology, although *Corynetis* is believed to have been a predatory organism with a burrowing lifestyle, possibly capable of constructing deeper vertical burrows [28], the detailed and systematic research on *Corynetis* remains limited.

Here, we conducted research on new materials from the Guanshan Biota and discovered *C. brevis* in it for the first time. By analysing and comparing the spiny ornamentations of *C. brevis* and *C. fortis*, a more detailed burrowing lifestyle and a new hunting pattern based on the sensory system have been established. Our results indicate that spiny ornamentations of *Corynetis* show simple structures, with the densely spinose morphology potentially reflecting a more sedentary, burrowing lifestyle.

## 2. Material and methods

### 2.1. Fossils

A total of 223 specimens of Corynetidae were discovered in the Guanshan Biota, among which 13 specimens of *C. brevis* and 25 specimens of *C. fortis* were precisely identified. All specimens were excavated from Longbao Mountain, Gaoloufang Village, Kunming City, Yunnan Province, China. The fossils were preserved in yellow-green mud shale of the Wulongqing Formation.

Specimens were initially examined under a Carl Zeiss Stemi 508 microscope, with detailed images captured using the associated instrumentation. Overall images were obtained using a Canon EOS 5D Mark IV camera. For more detailed morphological and compositional analysis, a Scanning Electron Microscope (SEM; Helios G4 UC) was employed. Specimens intended for SEM analysis were first affixed with conductive adhesive and then placed within the vacuum chamber of machine. Vacuuming was terminated once the chamber reached the required parameters (TMP1 = 99%; HVG ≤ 6**$e^{-3}$**). Secondary electron and backscattered electron images were acquired at an accelerating voltage of 3 kV and a beam current of 0.2 nA. These imaging techniques were complemented by Energy Dispersive Spectrometry (EDS) for elemental analysis. For EDS, the accelerating voltage was adjusted between 15 kV and 25 kV (with 20 kV being most commonly used) and the beam current was set to 3.2 nA, with the electron beam turned off. A new analysis region was then defined, scanning of images and collection of distribution map data were performed sequentially. The scanning results were monitored in real time via the distribution map data interface. Finally, Adobe Photoshop CS3 software was used for creating illustrations and formatting images. All specimens are housed in the Laboratory of Early Life and Environments at Northwest University.

### 2.2. Phylogenetic analysis

The dataset is used from Shi *et al.* [49] and Wang *et al*. [54]. We added a new genus *Anningvermis* [28] to the data matrix and modified the characteristics of *Corynetis*, *Sicyophorus* [18,30] and *Louisella* [39,52,55] (see electronic supplementary material). The final data matrix consists of 97 taxa and 180 characteristics (see electronic supplementary material), with *Acosmia* as the outgroup. Parsimonious analyses are performed with TNT v.1.5 [56], selecting New Technology Search, the test run under equal and implied weights [57,58], finally generated a consensual tree. Maximum parsimony analyses are performed with PAUP v. 4.0, setting maxtrees to 1000 in heuristic search, adjusting the swapping algorithm to TBR in branch swapping, all characters are of the type ‘unordered’ and have equal weight. Bootstrap analysis was set to nReps = 1000, ConLevel = 50.

The phylogenetic trees are adjusted in FigTree v. 1.4.4 and CorelDRAW 2021, combined with Adobe Photoshop CS3 to complete the graphics.

## 3. Systematic palaeontology

Phylum Priapulida Delage & Herouard, 1897
Class, Order uncertain
Family Corynetidae Huang, Vannier & Chen, 2004
Genus *Corynetis* Luo & Hu, 1999

*Type species. Corynetis brevis* [51]

*Other species. Corynetis fortis* [10]

*Emended diagnosis*. Club-shaped priapulid. Circumoral crown and coronal spines are two types of scalids arranged on the introvert. Circumoral crown is in the shape of short conicals around the mouth, with two circles, eight members per circle. Coronal spines present as a circle of strongly developed long spines, distributing at the margin of the introvert. The collar appears a smooth surface. A long pharynx covers with dense teeth. The trunk is densely covered with annulations, on which are arranged circles of spines. Each spine shows a broad base and sharp tip (modified from Hu *et al.* [10]).

*Localities and horizon*. Ercaicun village and Shankoucun village; Kunming City, Yunnan Province, SW China; Cambrian Series 2, Stage 3, Yu’anshan Formation. Gaoloufang village and Gangtoucun village; Kunming City, Yunnan Province, SW China; Cambrian Series 2, Stage 4, Wulongqing Formation.

*Corynetis brevis* Luo & Hu, 1999
Figures 1*h–p*, 2 and 3

**Figure 2.**
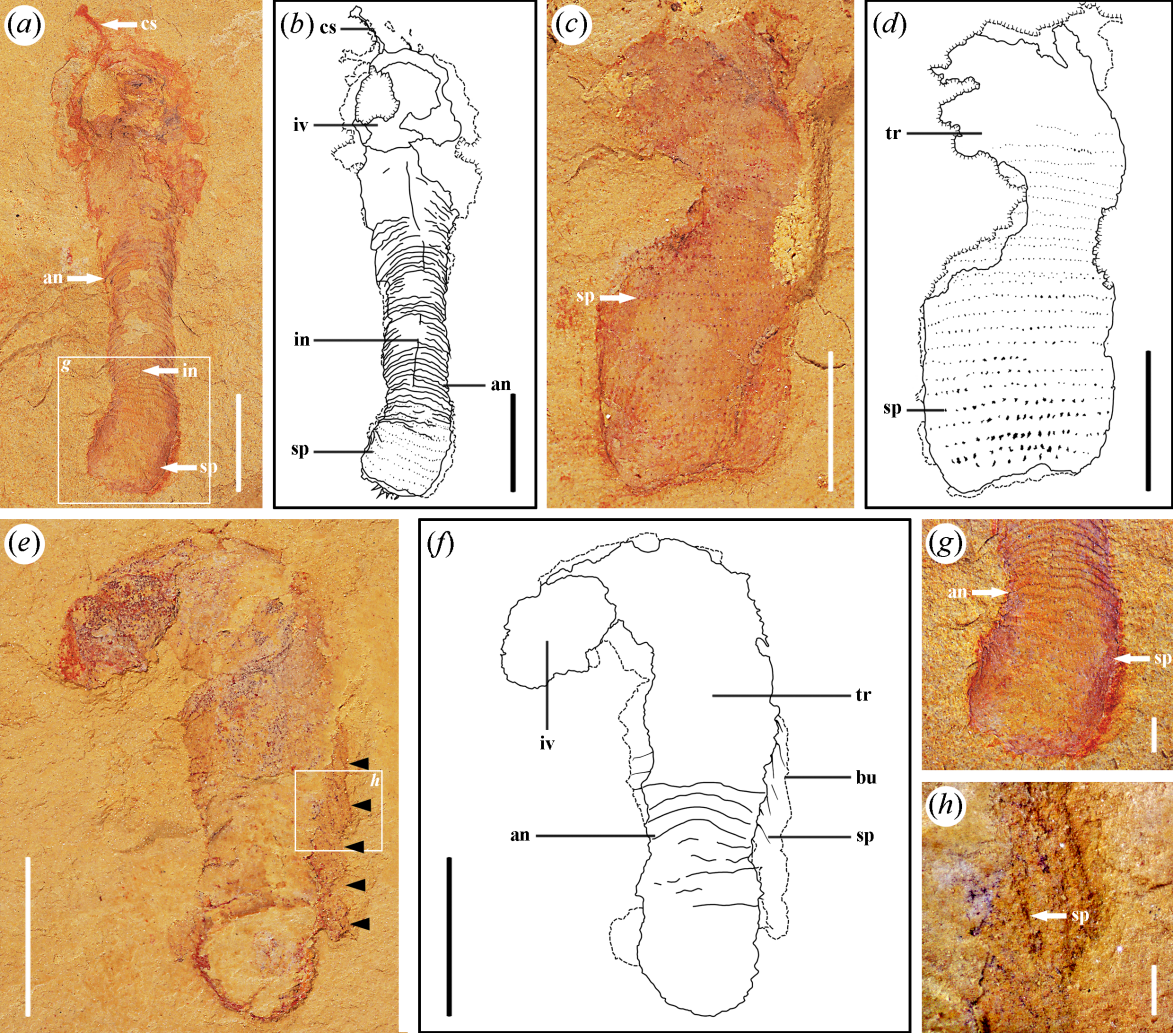
*Corynetis brevis* from the Guanshan Biota. (*a, b, g*) LBS−372; (*a*) Part, specimen has typical structures such as coronal spines (cs), annulations (an), intestine (in) and spines (sp); (*b*) Illustration of the part; (*c, d*) LBS−842; (*c*) Part, specimen with obvious spines (sp) on the trunk; (*d*) Illustration of the part; (*e, f, h*) LBS−520; (*e*) Part, specimen has spines (sp) at the lateral and pointing downwards. The black arrows indicate the boundary of burrow; (*f*) Illustration of the part; (*g*) Enlarged image of terminal trunk within the boxed area in (*a*), showing spines (sp) at the terminal; (*h*) Enlarged image of spines (sp) pointing downwards within the boxed area in (*e*). Abbreviations: an, annulations; bu, burrow; cs, coronal spines; in, intestine; iv, introvert; sp, spines; tr, trunk. Scale bars: (*a−f*), 5 mm; (*g*), 1 mm; (*h*), 0.5 mm.

**Figure 3.**
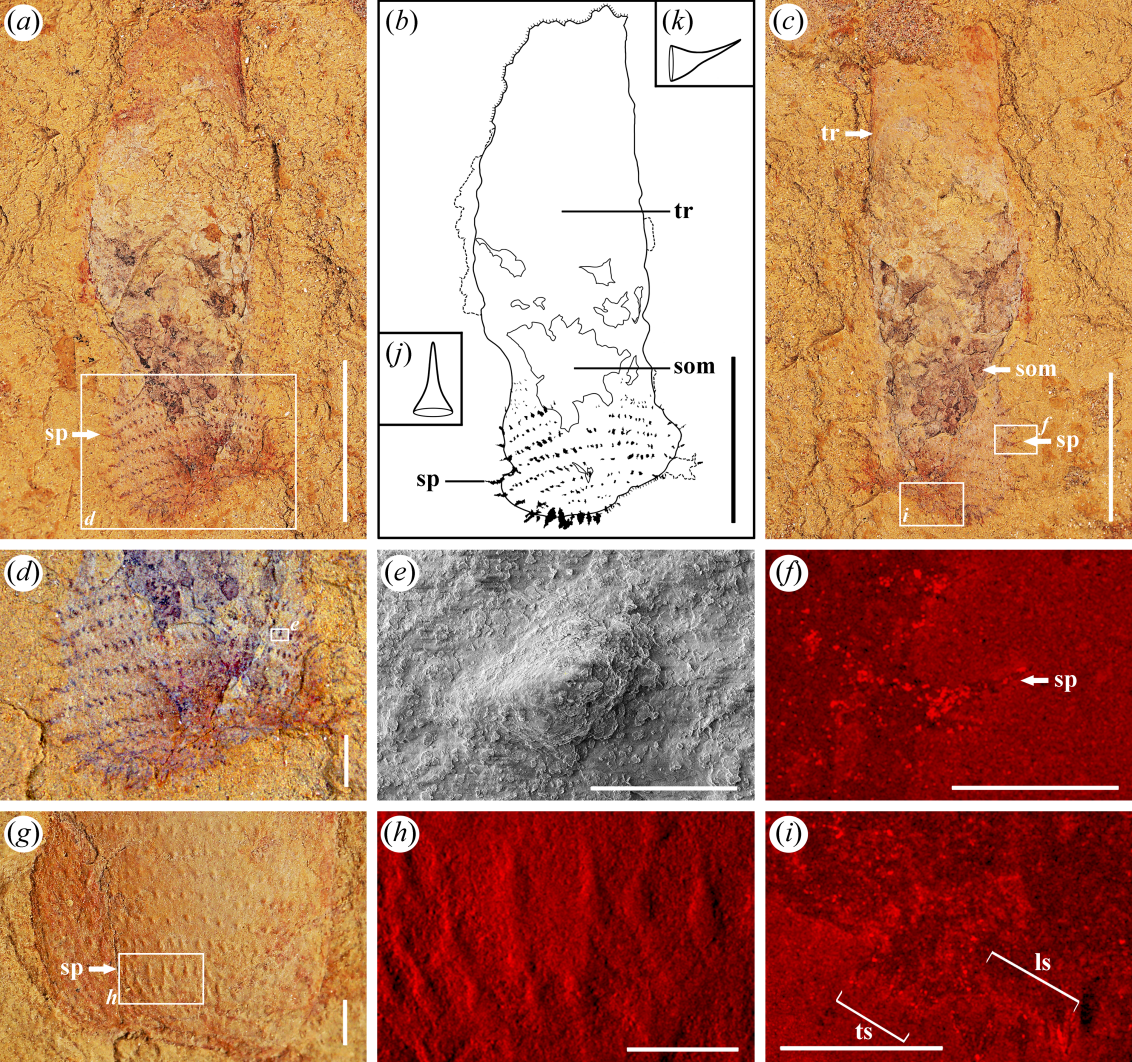
*Corynetis brevis* from the Guanshan Biota with well-preserved terminal trunk. (*a−f, i*), LBS−384; (*a*) Part, specimen with spines (sp) at the terminal; (*b*) Illustration of the part; (*c*) Counterpart; (*d*) Enlarged image of terminal trunk within the boxed area in (*a*), showing the details of spines (sp); (*e*) SEM image of spines (sp) on the trunk within the boxed area in (*d*); (*f*) EDS image of Fe element within the boxed area in (*c*), showing the detailed morphology of spines (sp) at the lateral; (*g, h*) LBS−842; (*g*) Enlarged image of terminal trunk; (*h*) EDS image of Fe element within the boxed area in (*g*), showing the detailed morphology of spines (sp) on the trunk; (*i*) EDS image of Fe element within the boxed area in (*c*), showing the morphology of the last circlet of spines (sp); (*j-k*) Illustration of the spines (sp) of *Corynetis brevis* on the terminal trunk. Abbreviations: ls, large spines; som, suspected organic matter; sp, spines; tr, trunk; ts, tiny spines. Scale bars: (*a−c*), 5 mm; (*d, g*), 1 mm; (*e*), 40 μm; (*f, h, i*), 500 μm.

*Diagnosis*. Trunk can be divided into three parts according to the length and direction of spines on it. The terminal trunk is significantly enlarged, and the spines on it are also bigger. Each of the spines on the terminal trunk shows an uneven contraction. In rare cases, a short caudal projection is preserved at the terminal end.

**Figure 4.**
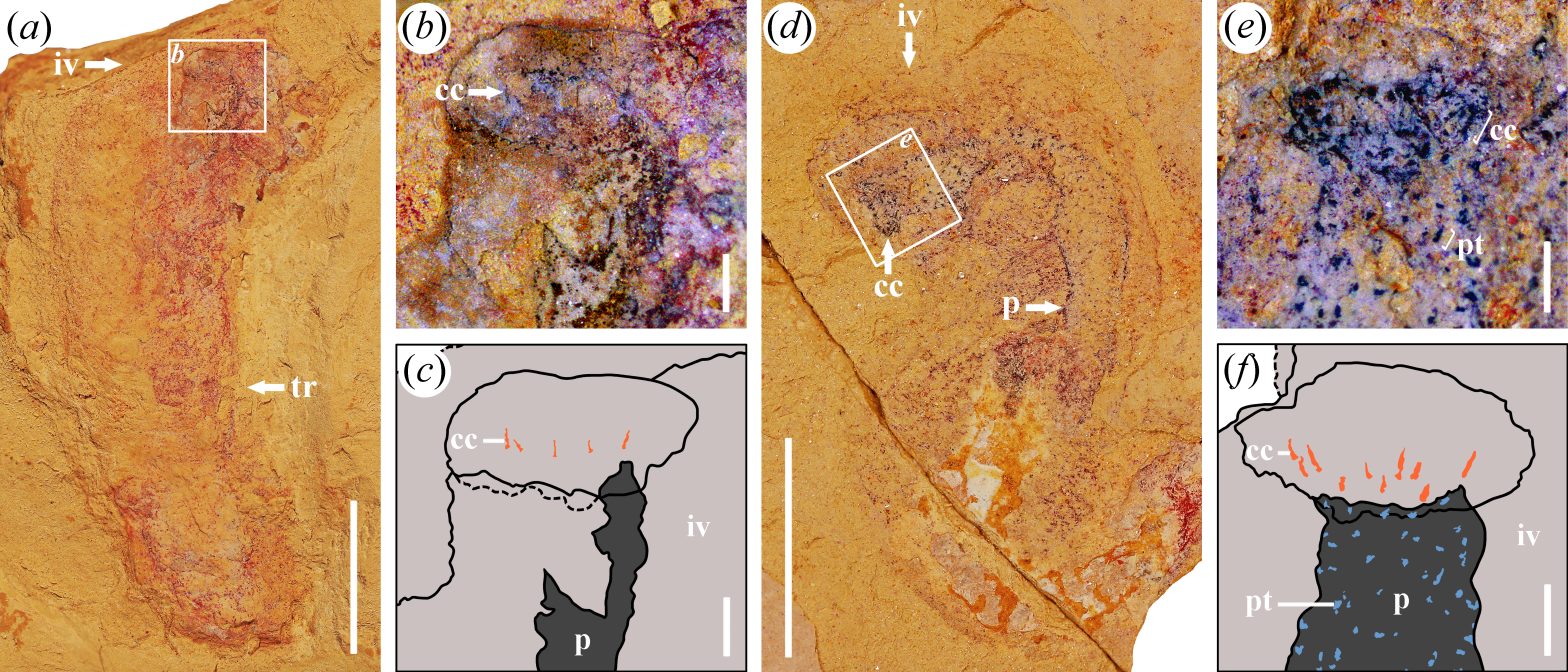
Circumoral crown of *Corynetis* in the Guanshan Biota. (*a−c*), LBS−491; (*a*) Part; (*b*) Enlarged image of circumoral crown (cc) within the boxed area in (*a*), the number of 5 can be seen in a single circlet on one side; (*c*) Illustration of (*b*); (*d−f*), LBS−681; (*d*) Part, specimen has typical structures such as circumoral crown (cc), pharynx (p) and pharyngeal teeth (pt); (*e*) Enlarged image of circumoral crown (cc) and pharyngeal teeth (pt) within the boxed area in (*d*). Circumoral crown (cc) can be seen in two circles, the number of each circle is about 5 on one side; (*f*) Illustration of (*e*). Abbreviations: cc, circumoral crown; iv, introvert; p, pharynx; pt, pharyngeal teeth; tr, trunk. Scale bars: (*a, d*), 5 mm; (*b, c, e, f*), 0.5 mm.

*Description*. There are 13 specimens, and 7 with preserved counterparts. The incomplete specimens almost lack the anterior portion. Only two specimens preserved the signature coronal spines (figures 1*h–j,m,n* and 2*a,b*). Five specimens preserved the circumoral crown (figures 1*m,n* and 4). Six specimens preserved the terminal trunk exquisitely, four of which exhibit swollen morphology (figures 2*a,b,g* and figures 3a-d,3), while the remaining two gradually contract backward and present a caudal projection at the end (figure 1*o,p*). The measurements indicate that the length of the complete specimens is about 17–39 mm, the average width is 3.8–7.5 mm, and the common aspect ratio is circa 9 : 2.

The body can be divided into the proboscis, trunk and caudal projection. A smooth transition exists between the proboscis and the trunk, without a defined neck region (figures 1*h*,*i*,*k*,*l*, and 2*a*,*b*).

**Figure 5.**
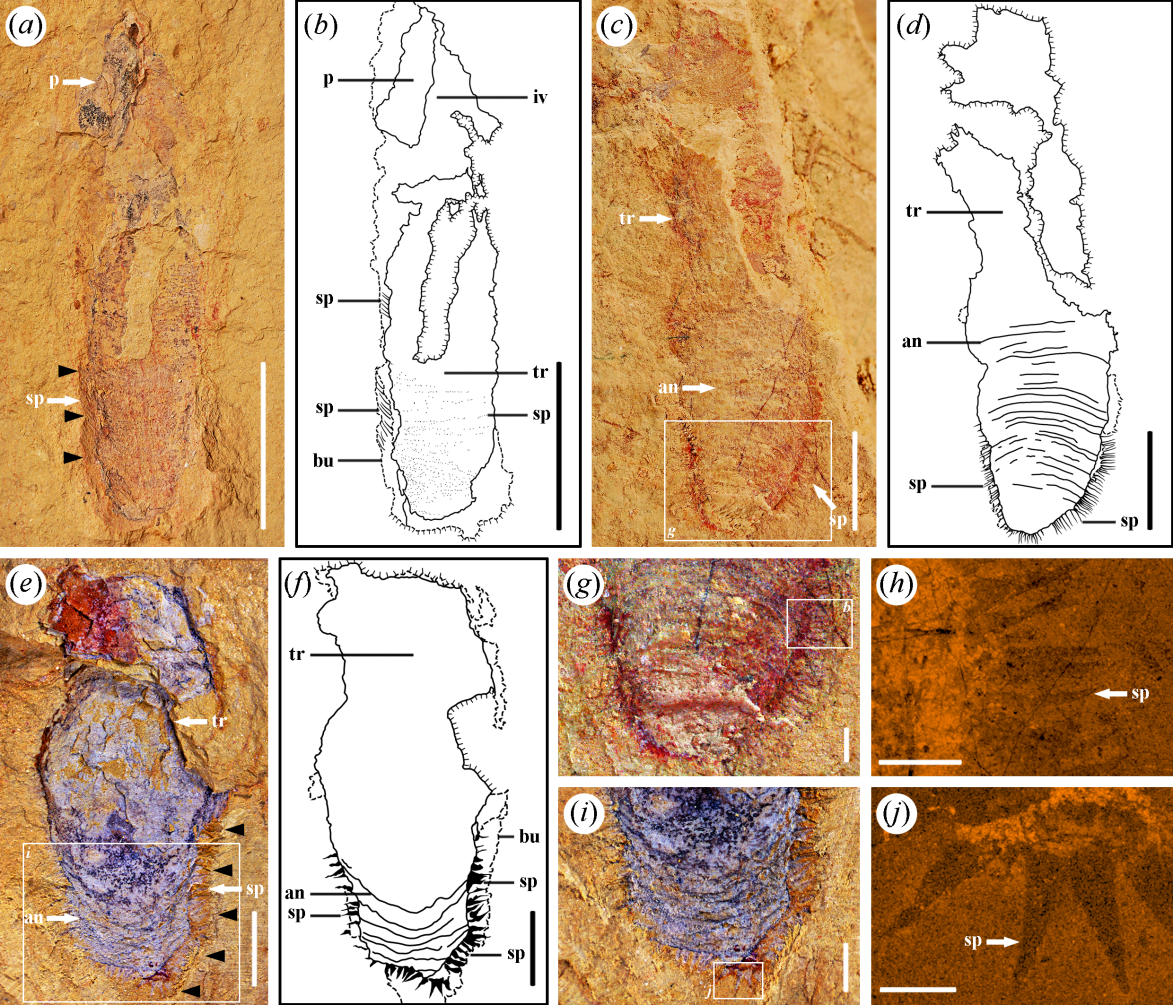
*Corynetis fortis* from the Guanshan Biota. (*a, b*), LBS−682; (*a*) Part, specimen preserved through layers, presenting typical structures of pharynx (p) and spines (sp). The black arrows indicate the boundary of burrow; (*b*) Illustration of the part; (*c, d, g, h*), LBS−706; (*c*) Part, specimen has typical structures such as annulations (an) and spines (sp); (*d*) Illustration of the part; (*e, f, i, j*), LBS−712; (*e*) Part, specimen with clear spines (sp) at the terminal trunk. The black arrows indicate the boundary of burrow; (*f*) Illustration of the part; (*g*) Enlarged image of terminal trunk within the boxed area in (*c*), showing morphology of spines (sp); (*h*) EDS image of Fe element shows the detailed morphology of spines (sp) within the boxed area in (*g*); (*i*) Enlarged image of terminal trunk within the boxed area in (*e*), showing morphology of spines (sp); (*j*) EDS image of Fe element shows the detailed morphology of spines (sp) within the boxed area in (*i*). Abbreviations: an, annulations; bu, burrow; p, pharynx; iv, introvert; sp, spines; tr, trunk. Scale bars: (*a−d*), 5 mm; (*e−g, i*), 1 mm; (*h*), 500 μm; (*j*), 250 μm.

The proboscis comprises three parts from posterior to anterior: introvert, collar and pharynx. Introvert is uniform in width, or slightly narrow forward (figure 1*h,i*). The introvert surface has scalids, including the circumoral crown and coronal spines (figure 1*m*,*n*). The circumoral crown is distributed around the mouth and shape in cone, showing two circles in total, with eight scalids in each circle (figure 4*b*,*c*,*e*,*f*). Coronal spines are preserved as elongated, linear structures, with a mineralized surface that appears reddish in colour (figures 1*m,n* and 2*a,b*). The collar is characterized by a smooth surface and a forward tapering morphology. The pharynx is long and slender (figures 4 d and 5a,b), exhibiting a high degree of flexibility that allows it to be twisted at multiple angles, and it may appear either straight or curved. Typically, the pharynx is internal to the body (figure 4*d*), although it could evert externally, where it is likely to rupture under pressure (figure 5*a,b*). The acicular pharyngeal teeth are arranged in oblique rows on the pharyngeal surface (figure 4*d–f*). The pharynx is generally preserved in a bluish-black colour, which is darker than the surrounding body tissue. In contrast, the pharyngeal teeth are preserved in a more distinct, aterrimus-like colour.

The trunk is usually uniform in width, but has a marked swelling at the terminal end (figures 2*a*,*b* and 3*a-*c). The surface of the trunk develops dense annulations (figure 2*a,b*). Annulations are barely visible at the front of the one-sixth portion, then become clear towards the posterior, and gradually disappear at the swollen portion (figure 2*a,b*). Abundant circles of spines are also arranged on the trunk, adjacent circles are arranged in a staggered manner (figures 2*a-d* and 3*d,g*). According to the length and direction of the spines, the trunk can be divided into three regions. Trunk I exhibits longer spines pointing backward. Trunk II shows shorter spines pointing backward (figure 2*e,f*,*h*). Trunk III presents a swollen form, the spines on which enlarge markedly (figure 2*c,d,g* and 3*a–d*). Spines on trunk III are flattened into a straight or slightly curved shape at the lateral (figure 3*d*) and presented as round or oval protrusions in the middle (figure 3*e*). SEM analysis shows that these spines whether distributed in the lateral or in the middle always present as cones and have a narrow tendency towards the distal end (figure 3 *f, h*). Each of the spines tapers abruptly within the first third up from the base and maintains a slender form that slowly converges into a single tip (figure 3*j,k*). In the front four-fifths portion of trunk III, the spines are relatively slender and point forward (figure 3*d*). In the last fifth, the spines are thicker and point backward (figure 3*a,b,d*). The last row of spines are short and stout, and vary in size (figure 3*d*), five to six spines in the middle are smaller, and the lateral spines are thicker (figure 3*i*). On the single side of the adult specimens, 26–27 trunk spines are visible per circle (figures 2*a–d* and 3*d,g*). Thus, there are approximately 53 spines per circle in the adult phase. A few specimens show the maximum width at the middle trunk, contracting backward and appearing a caudal projection at the terminal end (figure 1*h,i*, *k,l*). Caudal projection smoothly connects to trunk, and no boundary between them is found (figure 1*o,p*). The surface of the caudal projection is unadorned.

The intestine is preserved as a flat or slightly curved tube along the central axis of the trunk, with a greyish-black colour (figure 2*a,b*).

*Localities and horizon*. Gaoloufang village; Kunming City, Yunnan Province, SW China; Cambrian Series 2, Stage 4, Wulongqing Formation.

*Corynetis fortis* Hu et al., 2012
Figure 5

*Diagnosis*. The trunk is consistent in width, with a tapering morphology at the posterior end. Spines on the trunk point forward, with a directional change only at the last few circles. Each of the spines on the terminal trunk exhibits uniform constriction.

*Description*. There are 25 specimens and 18 with preserved counterparts. The incomplete specimens are mostly preserved as trunk fragments, 21 of which have well-preserved spines (figures 5,5). Measurements show that the length of adult specimens is approximately 20–41 mm, and the average width is approximately 5–9 mm.

The body consists of proboscis and trunk, without neck (figure 5*a*,*b*). The morphology of the proboscis is similar to that of *C. brevis*.

The trunk is uniform in width, narrowing only in the last tenth portion at the terminal end (figure 5*c*,*d*). Dense annulations are arranged on the trunk (figure 5*c-f*). Circles of spines follow the annulations. The adjacent rings of spines are in a staggered array (figure 5*a*,*b*). Spines primarily point forward (figure 5*a*,*b*), only the last few circles change direction to point backward (figure 5*c–g*,*i*). Each of the spines on the terminal trunk is shaped as a cone, uniformly tapered from the base to the tip (figure 5*h,j*). The length-to-width ratio of the terminal spines is 3 : 1 to 5 : 1 (figure 5*h,j*). All the spines wrap tightly around the trunk. Caudal projection or caudal appendages are not identified.

*Remarks*. Five specimens preserve the peri-oral structure, characterized by short spines arranged in circlets, which are referred to as the circumoral crown (figure 4). Based on the observation of 4–5 spines visible on one side (figure 4*b*,*c*), the number of spines per circlet is estimated to be approximately eight. The circumoral crown of specimen LBS−681 is well preserved and exhibits a staggered row arrangement (figure 4*e*,*f*), suggesting the presence of two concentric circles. To minimize the potential interference from adjacent spiny ornamentations, comparative analyses were conducted. By comparing with the pharyngeal teeth below, it can be proved that the circumoral crown is nearly twice as long as the pharyngeal teeth (figure 4*e,f*). In addition, the three-dimensional arrangement of these structures is not fully preserved due to taphonomic compaction, which results in overlapping features. However, in specimens that preserve coronal spines, the circumoral crown is faintly visible in a position closer to the mouth than the coronal spines (figure 1*m,n*), indicating a distinct spatial relationship. This observation supports the conclusion that the circumoral crown is not a remnant of broken-off coronal spines, but rather a distinct and coexisting morphological feature.

**Figure 6.**
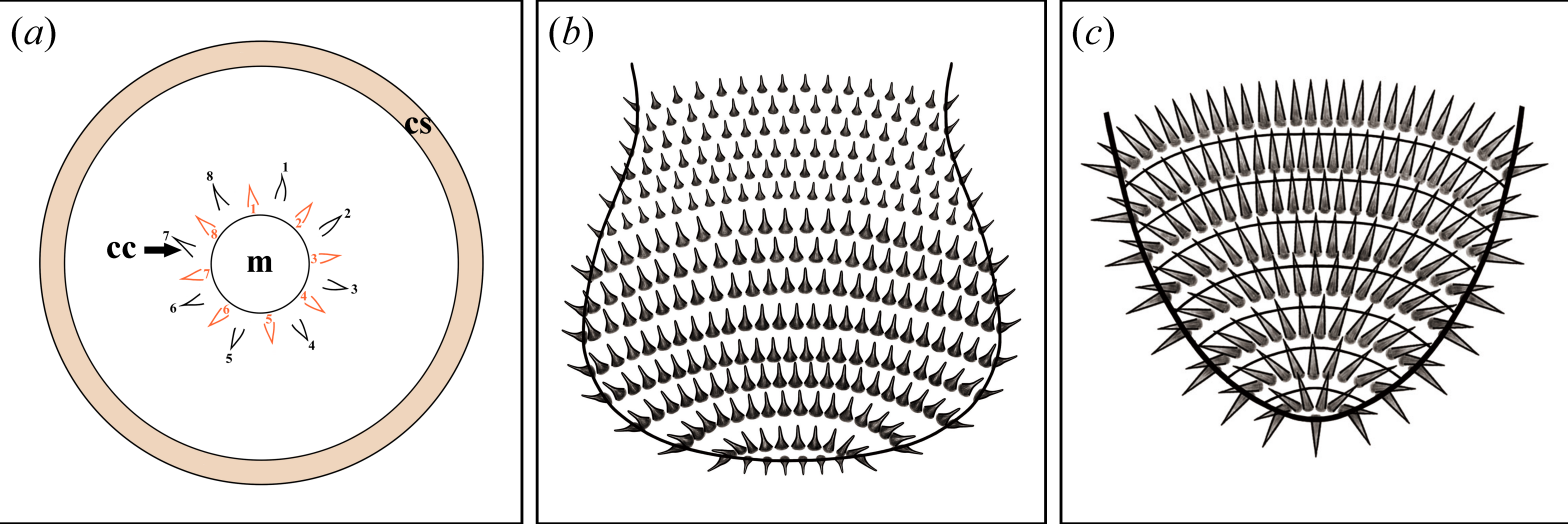
Illustrations of spiny ornamentations of *Corynetis*. (*a*) Illustration of scalids. Orange scalids represent the first circle of circumoral crown (cc), the black represent the second circle, the number of each circle is 8. The coloured part represents the distribution band of coronal spines (cs); (*b*) Illustration of the terminal trunk of *Corynetis brevis*; (*c*) Illustration of the terminal trunk of *Corynetis fortis*. Abbreviations: cc, circumoral crown; cs, coronal spines; m, mouth.

Furthermore, it is concluded that the circumoral crown represents a supplementary morphological character of *Corynetis*, and should therefore be included in the generic diagnosis. A schematic diagram illustrating the distribution of scalids in *Corynetis* is deduced (figure 6*a*).

*Localities and horizon*. Gaoloufang village; Kunming City, Yunnan Province, SW China; Cambrian Series 2, Stage 4, Wulongqing Formation.

## 4. Discussion

### 4.1. Spiny ornamentations

The introvert of *Corynetis* has undergone a marked specialization. The scalids on the introvert of *Corynetis* possess two distinct types: the circumoral crown and coronal spines. These scalids are sparsely distributed, forming only three circlets. In contrast to other priapulid taxa such as *Xiaoheiqingella* [33,50,59], *Yunnanpriapulus* [50], *Tylotites* [52], *Sicyophorus* [18,30], *Ottoia* [38,40], *Paraselkirkia* [7], *Ercaivermis* [54], *Eximipriapulus* [36,53] and *Xiaolantianella* [60], which exhibit abundant scalids and cover half or even the entirety of the introvert. It is undoubtedly a remarkable distinction. Therefore, it is hypothesized that the first circlet of eight scalids of *Corynetis* represents a symplesiomorphic trait [54,61,62], while a certain circlet or the last circlet grows rapidly, evolving into strongly developed coronal spines, with the remaining scalids degenerating over time.

The introvert of *Corynetis* is unlikely to serve as an anchor to assist movement, but the scalids on it have a sensory function. In extant *Priapulus caudatus*, its introvert undergoes acute deformation during movement, with scalids acting as terminal anchors to facilitate forward locomotion [63,64]. In contrast, fossils show that the introvert of *Corynetis* has limited deformability. In addition, the circumoral crown is short and sparse around the mouth, making it unsuitable for substrate contact or anchoring. The coronal spines, while fine and needle-like, have an exaggerated aspect ratio, which also precludes an anchoring function. In addition, the scalids arrangements of extant *Priapulus* and *Priapulopsis* are significantly different from *Corynetis* [65], while that in *Acanthopriapulus horridus* is similar to *Corynetis. A. horridus*, a rare extant species, has densely arranged scalids at the anterior, which gradually diminish towards the posterior [65]. Specifically, scalids around the mouth are small in number and irregularly arranged, but organize into 25 longitudinal rows towards the rear [65]. The tubular structures at the tips of *A. horridus* scalids suggest a sensory role [65]. Commonly in previous research, scalids in priapulids are not only cuticular structures but are also innervated by nerve clusters [66–68]. The first circlet of scalids is considered to have a sensory function [54]. Therefore, both the circumoral crown and coronal spines of *Corynetis* are interpreted as having sensory functions, with the specific role of the former remaining unclear and the latter playing a key role in predation. All the scalids have served a defensive purpose.

Unlike the introvert, trunk spines of *Corynetis* retain an anchoring function, forming a single-anchor strategy in withdraw locomotion. Similar to *Corynetis*, *Tylotites petiolaris* in the Chengjiang Biota also bears trunk spines [52]. These spines are thought to aid locomotion through the coordination of longitudinal muscles [64], which supports the idea that the spines of *Corynetis* also played a role in movement. The morphology of spines on the terminal trunk of *C. brevis* and *C. fortis* differs (figures 3 *j,k* and 5*j*), which may be related to the distinct terminal trunk morphologies (figures 6b,c,6,6). In *C. brevis*, the terminal trunk appears enlarged when body fluids are aggregated elsewhere (figures 2*a,b* and 3*a,b*), while in *C. fortis*, the terminal trunk is narrow when the trunk is uniform in width (figure 5*c,d*). This suggests that *C. brevis* may rely primarily on the terminal trunk for anchoring and movement, whereas *C. fortis* uses the entire trunk for this purpose. These single-anchor structures are composed of spines pointing forward, which facilitate the backward retracting of *Corynetis*, but not for advancing forward. Furthermore, *C. brevis* is likely to possess at least two types of trunk spines with distinct functions. While only some serve an anchoring role, all trunk spines appear to have a defensive role.

The spiny ornamentations of *Corynetis* in the Guanshan Biota appear to be in an early stage of evolution, with a relatively simple morphology. The trunk spines of *Corynetis* and *T. petiolaris* in the early Cambrian are morphologically simple, but differ in curvature and constriction. While compared to the spiny ornamentations of *Ottoia* in the middle Cambrian, more complex structures, such as the denticles and spurs appear [40]. In extant *Meiopriapulus fijiensis*, there are more elaborate features, including distal elements, bifurcated structures, tubular projection and fibrillar bands [69].

### 4.2. Palaeoecology

*Corynetis* in the Guanshan Biota shows an endobenthic lifestyle, with the exceptional preservation of fossil materials. The Guanshan Biota is a classic example of a Burgess shale-type Lagerstätten [13,34,43,70,71], classified as a Tier 2 deposit [71,72]. The Burgess shale-type preservation is a taphonomic mode preserving soft-bodied fossils, typically through rapid burial in fine-grained sediments under anoxic conditions [72–74]. The preservation style is often associated with short-distance transport and rapid burial, with carbonate cements forming an early sealing environment that aids in the preservation of organic tissues as thin carbon films [72–74]. In the Guanshan Biota, sedimentological and taphonomic evidence suggests that its depositional environment was shallower and closer to the continental sources than the other typical Burgess shale-type deposits [11–13,71,75–78], indicating a nearshore setting [13,76,77,79,80]. Soft-bodied fossils are preserved primarily through rapid burial by distal storm flows [11,34,43,75,76], and the presence of pyrite supports the interpretation of anoxic conditions [12,72,80,81]. Meanwhile, evidence of *in situ* burial is also provided: (i) Biological interactions—fossils of palaeoscolecidomorphs, brachiopods, parasitic tube worms, arthropods are preserved in mutually overlapping layers [76,82]. Clusters of hyoliths showed non-directional arrangements and variable preservation depths [80]. This evidence suggests *in situ* assemblages [76,80,82]. (2) Biological disturbance—horizontal traces and vertical excavations indicate minimal transport [11,75,76,79,82]. (3) Biological preservation—hyoliths retain the connection between the soft part and hinge structure [80]. Ten *Corynetis* specimens in our collection preserve both soft tissues and burrowing traces, strongly supporting *in situ* burial (figures 2*e,f* and 5*a,b*,*e,f*).

**Figure 7.**
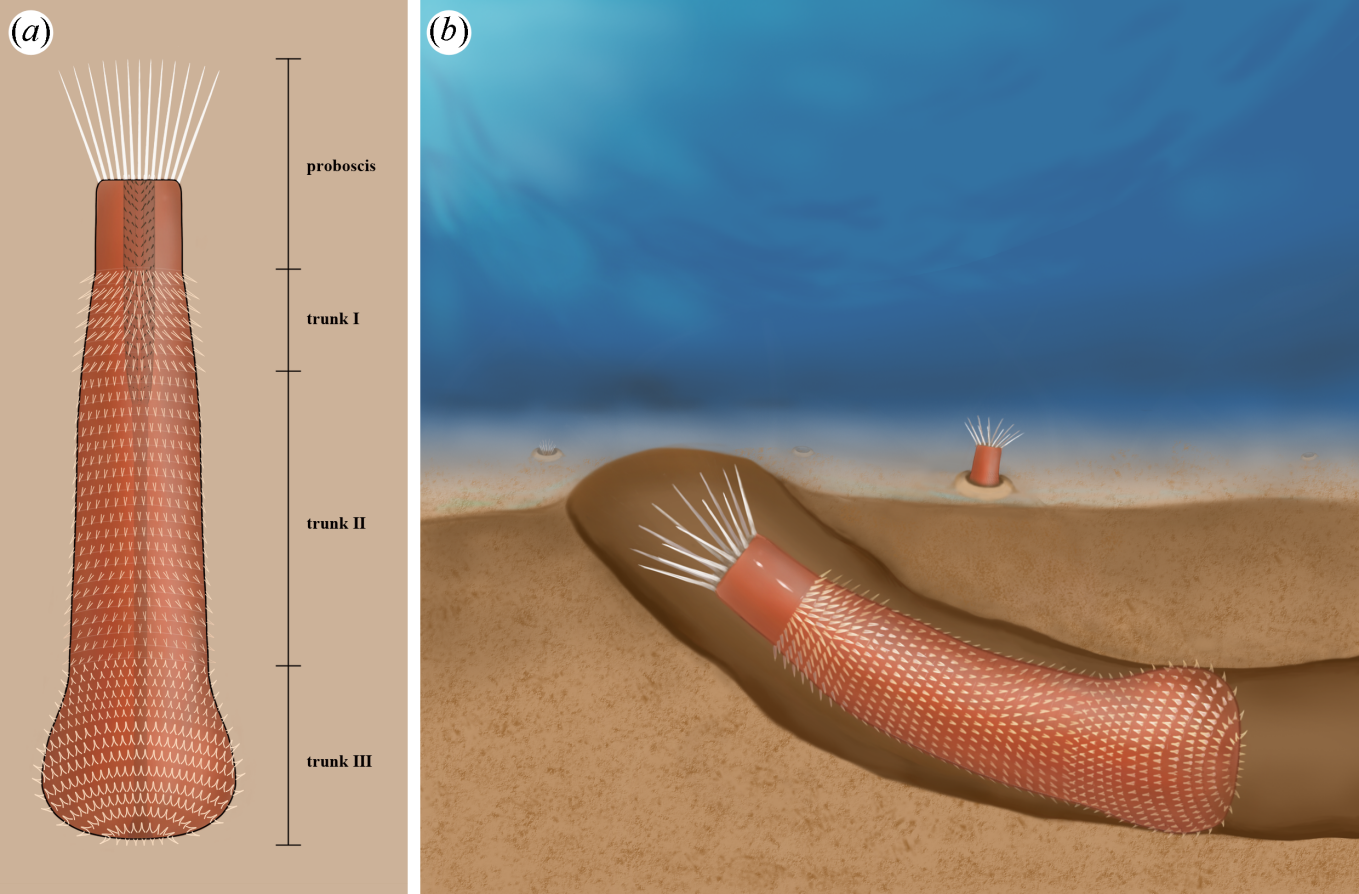
Reconstructed images. (*a*) Reconstructed image of *Corynetis brevis*; (*b*) Ecological reconstruction.

*Corynetis* shows a solitary lifestyle, relying on the protection of burrows to carry out its life activities. In our collection, *Corynetis* fossils are generally isolated, with few associated vermiform taxa. Their flexible pharynx, strong coronal spines and spinose trunk indicate strong predatory and defensive capabilities, enabling *Corynetis* to thrive in a competitive environment. In addition, *Corynetis* is commonly preserved in sedimentary layers, with some specimens in burrowing positions (figures 2*e,f* and 5*a,b,e,f*) [28]. The terminal trunk is often buried in lower layers (figure 5*a,b*), suggesting deeper burrowing (figure 7*b*). *Corynetis* is supported by a hydrostatic skeleton and burrows via peristalsis. The abundance of forward-pointing spines on the trunk is not conducive to active burrowing, suggesting that *Corynetis* spent most of its time hidden in burrows, only emerging briefly for essential activities. *Corynetis* grasps the wall of the burrow tightly by the anchoring function of the trunk spines, enabling itself to stabilize and rapidly retreat into the burrow, making the movement more flexible. When *Corynetis* is partially exposed, the coronal spines may serve as both a sensory and a defensive mechanism. When fully exposed, the trunk spines provide additional protection (figures 7,7).

*Corynetis* shows a carnivorous lifestyle, and the sensory function of coronal spines greatly enhances the predatory efficiency. The coronal spines could actively expand outwards, and when they radiate out in the environment, the opportunities to encounter prey are increased. Once *Corynetis* sense prey by physical contact or environmental cues (water currents, chemical signals and temperature gradients), they could reach out their elongated pharynx to capture the prey instantly. Then the pharynx retracts and *Corynetis* rapidly moves backward to drag the prey back to the hole, where the prey suffocates to death.

### 4.3. Phylogeny

**Figure 8.**
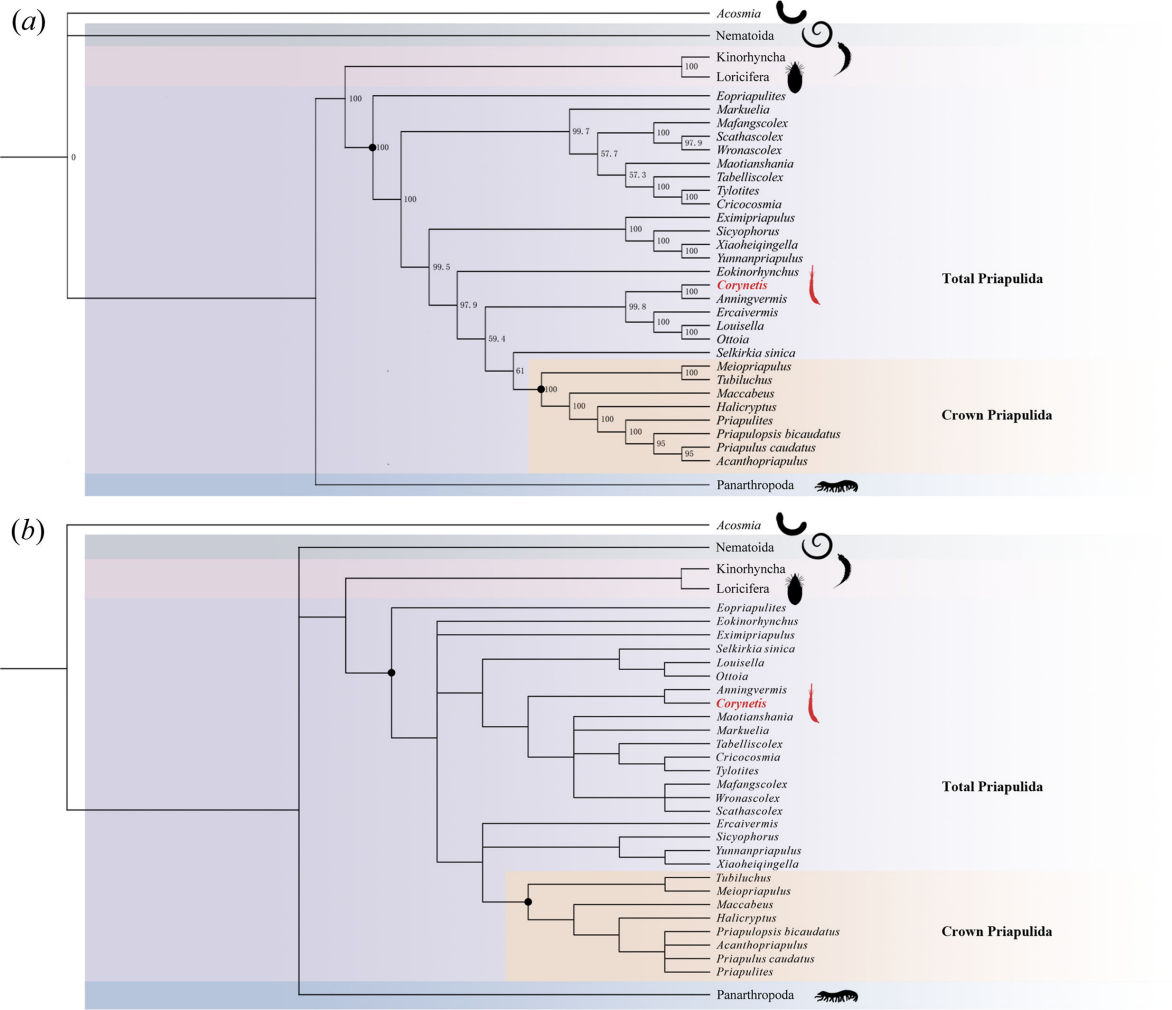
Phylogenetic trees. (*a*) Majority consensus tree by PAUP (1000 MPTs; tree length = 210.78; consistency index = 0.795; retention index = 0.932); (*b*) Parsimonious analyses by TNT. The red marks indicate the genus mentioned in the text.

The phylogenetic analyses are based on updates to detailed characteristics, especially our research on the spiny ornamentations of *Corynetis*, which modified 12 character codings (see electronic supplementary material), refining its phylogenetic position. In this study, the new morphological dataset mainly showed that the introvert of *Corynetis* has a total of three circles of scalids, and the first contains eight elements, with an unarmed surface at the distal end (the part close to the trunk), in addition to the circlets of spines present at the terminal region of the trunk. The results of the phylogenetic analyses by PAUP (figure 8*a*) and TNT (figure 8*b*) both place *Corynetis* within the stem-group Priapulida, recover *Corynetis* and *Anningvermis* as a monophyletic group. For other genera, our phylogenetic results strongly support that *Xiaoheiqingella*, *Yunnanpriapulus* and *Sicyophorus* are related taxa, that *Tabelliscolex*, *Tylotites*, *Cricocosmia* form a clade, and that *Louisella* and *Ottoia* are a monophyletic group. The position of *Ercaivermis* is always close to the crown-group Priapulida.

*Corynetis* is a stem-group priapulid and forms a monophyletic group with *Anningvermis*, which is consistent with the previous results [19,53,83–85]. Both *Corynetis* [10,28,51] and *Anningvermis* [28] have a similar three-part proboscis and spinose trunk, especially the coronal spines and trunk spines, which are uncommon and may be transitional characteristics in evolution.

*Louisella* has a more distinct relationship to *Corynetis* than *Anningvermis* [19,53,84,85]. *Louisella* possesses a ring of long spines on the introvert [39,86], similar to the coronal spines of *Corynetis*. In previous studies, only the genera of *Corynetis* [10,28,51], *Anningvermis* [28] and *Louisella* [39] possess coronal spines and were compared in phylogenetic analysis [83,84]. However, *Louisella* exhibits distinct differences in pharyngeal and trunk morphology. Its pharynx is swollen at the distal end and unarmed in the middle [39]. The trunk bears two longitudinal ventral appendages [39,55]. These features support its placement distinct from *Corynetis* and *Anningvermis*. In addition, the result that *Louisella* and *Ottoia* have a closer relationship is in line with previous studies [44,87].

## 5. Conclusion

Research on early Cambrian priapulids is progressing steadily, yet there remains a significant gap in our understanding of the detailed morphology, ecological patterns and evolutionary trends of certain groups. *Corynetis*, a stem-group priapulid from the Guanshan Biota, provides new insights into the detailed spiny ornamentations and functional morphology of this group. The circumoral crown of scalids is identified on the introvert, representing a novel morphological feature. This structure has eight scalids in the first circlet, which preserves a symplesiomorphic trait, suggesting an ancestral condition. The terminal trunk spines of *C. brevis* and *C. fortis* exhibit distinct morphologies, indicative of divergent anchoring strategies: *C. brevis* likely used the terminal region for stability, whereas *C. fortis* relied on the entire trunk. Palaeoecological analysis suggests that *Corynetis* occupied endobenthic niches, with scalids serving a sensory function to enhance predation and trunk spines aiding rapid withdrawal in burrows. These features support its role as a solitary predator in the early Cambrian marine ecosystem. Phylogenetic analysis confirms *Corynetis* as a stem-group priapulid, closely related to *Anningvermis*, forming a monophyletic clade. This study contributes to a more detailed understanding of priapulid morphology and the reconstruction of early Cambrian ecosystems.

## Data Availability

All data are available. The electronic supplementary material has been uploaded to Figshare [88].
